# Bladder duplication in the male cat: the first case report in China

**DOI:** 10.1186/s12917-024-04178-6

**Published:** 2024-09-06

**Authors:** Mingyuan Li, Yuqing Deng, Haoqian Liu, Jiaxu Sun, Shaokang Hong, Chen Lu, Christopher R. Mannion, Marta Carreño Gútiez, Bo Liu, Feng Yu

**Affiliations:** 1 College of Veterinary Medicine, China Agricultural University, Beijing, China; 2 Gumei Branch of Shanghai Muai Pet Hospital, Shanghai, China; 3 Shanghai GlinX Biotechnology Co., Ltd, Shanghai, China; 4 College of Veterinary Medicine and Biomedical Sciences, Colorado State University, Fort Collins, CO USA; 5 Center for Animal Disease Modeling and Surveillance (CADMS), Department of Medicine & Epidemiology, School of Veterinary Medicine, University of California, Davis, Davis, CA USA

**Keywords:** Feline medicine, Bladder duplication, Urogenital anomaly, Diagnosis and treatment

## Abstract

**Background:**

Bladder duplication is a rare congenital lower urinary tract anomaly disease characterized by the presence of two bladders, possibly with duplication of the urethra. This disease is rarely reported in cats. The clinical symptoms are commonly occult, with increased difficulty in making a definitive diagnosis, especially if there is no obvious urethral duplication. The diagnosis is typically based on radiographs and ultrasound, with computer tomography serving as a more advanced imaging diagnostic modality. Cases of duplicated bladders with accessory tubular tissues are even scarcer in both human and veterinary medicine.

**Case presentation:**

A 6-year-old male neutered cat was brought to the hospital because of vomiting and constipation. Cystography revealed increased soft tissue density of a fusiform structure in the lower middle abdomen. The purulent-filled cavitary structure and the accessory tubular structure were removed via surgery, and histopathological examination confirmed a double bladder with attached accessory tubular tissue. After antibiotic treatment, the cat recovered uneventfully.

**Conclusion:**

This is the first case of bladder duplication in China and the first case of feline bladder duplication with tubular structure attachment in the world. This information will provide a reference for the diagnosis and treatment of similar cases in the future.

## Background

**Fig. 1:**
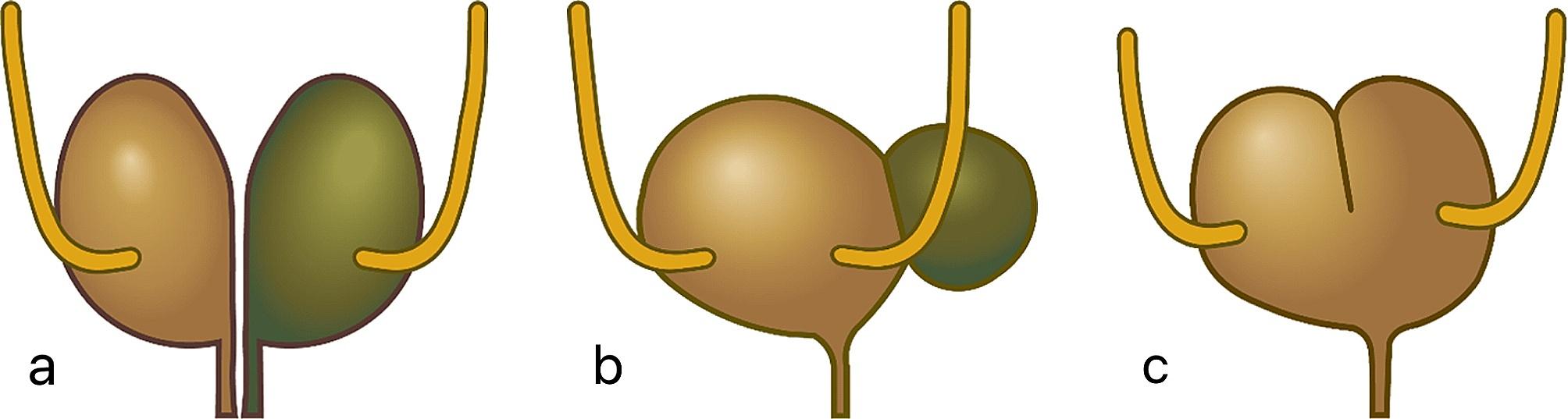
Bladder duplication diagram of sagittal duplication.**a**: Complete bladder duplication and urethral duplication. **b**: Complete bladder duplication. **c**: Incomplete bladder duplication

Bladder duplication (BD) is a congenital lower urinary tract anomaly that is rarely reported in both humans and animals [1, 2]. Bladder duplication can be classified into complete and incomplete forms based on different morphologies. In human medicine, two bladders are entirely separated by a normal bladder wall (mucosa, muscular wall, and peritoneal fold) in the complete bladder duplication form. In some cases, even the urethra is completely divided into two parts and connects to two separate bladders. In incomplete bladder duplication, the primary and accessory bladders may communicate and drain into a common urethra [2, 3]. Furthermore, bladder duplications can be categorized as sagittal duplications (more common, Fig. 1) or coronal duplications (less common). Sagittal duplication refers to the separation of the bladders along a plane parallel to the sagittal axis, while coronal duplication involves the separation of the bladders by an inclined muscle septum, with one bladder positioned in front of the other [4]. Congenital urinary malformations are quite infrequently observed in animals and are even rarer in cats; only one case involving a feline urinary malformation has been reported [5].

Elucidation of the underlying pathogenesis remains elusive both in human medicine and veterinary medicine. One hypothesis is ‘twinning of the embryo tail’ [6], and one suggested by Satter and Mossman named this the “double allantoic duct” [7]. Both of these findings indicated that duplications formed after the abnormal primordium developed during the foetal period. The embryogenesis of other duplicated organs illustrated that the development of uterine didelphys was due to the failure of the fusion of paramesonephric structures such as the Mullerian and Wolffian ducts [8].

Here, we describe the first case in China, review the literature on BD and analyse and summarize its clinical, diagnostic, and treatment options, aiming to provide valuable insights for future investigations on this particular condition.

## Case presentation

A six-year-old British shorthair cat, a neutered male, came to the veterinary hospital for chronic constipation and possibly frequent urination. In the last two days, the cat vomited frequently and had trouble defecating. The cat also had right-sided intra-abdominal cryptorchidism and had been neutered at the age of one. No other significant medical history was relevant. On physical examination, the patient was bright, alert, and responsive, his body condition score (BCS) was 3/5, and his vital signs were normal. A firm, round mass was palpable in the lower abdomen of the cat but no painful response was observed during palpation.

**Fig. 2:**
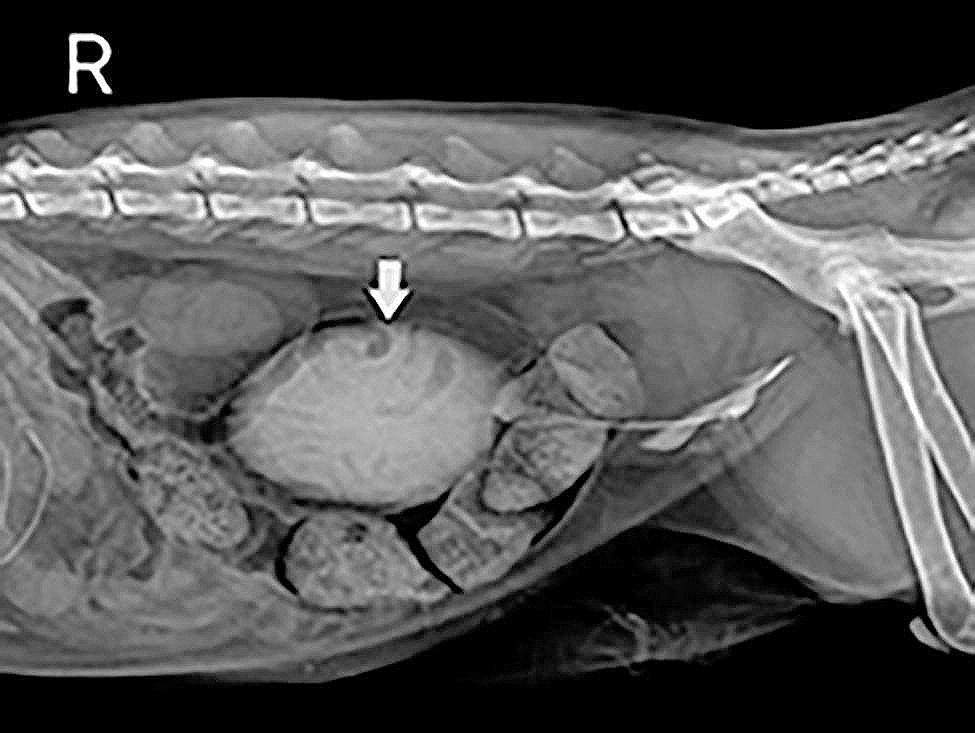
Cystography image of the cat. The bladder (arrow) near the mid-abdomen with contrast enhancement and a large amount of fecal material in the colon were observed

**Fig. 3:**
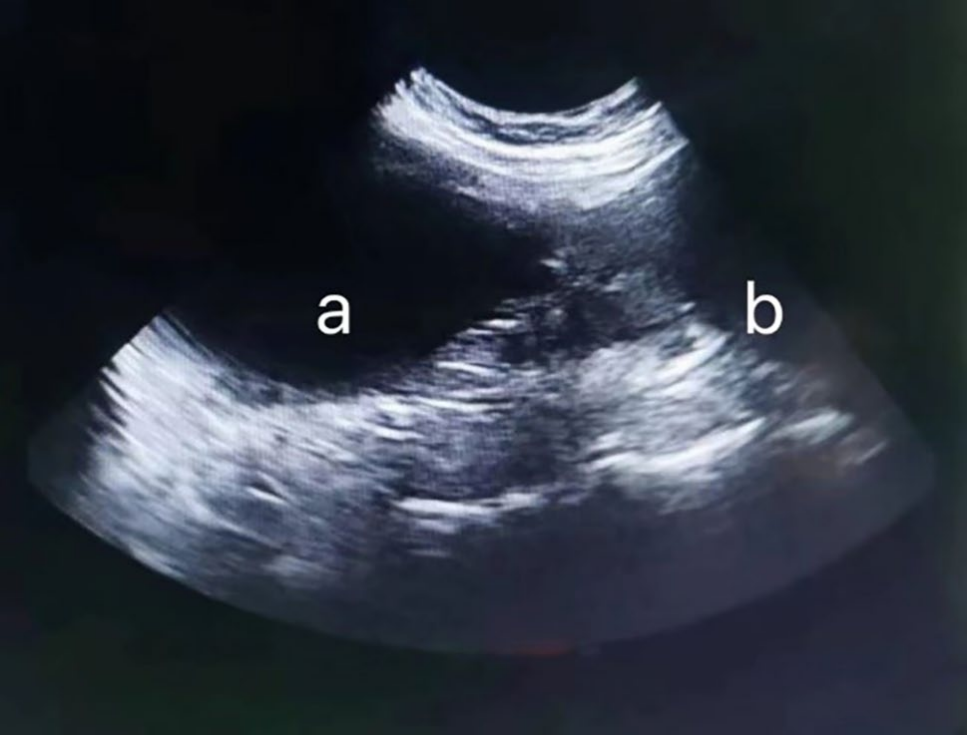
Ultrasonographic image of the cat. Two anechoic cystic structures were found in the abdomen, with no connection between them. **a** The cavitary structure was anechoic without obvious abnormalities, accompanied by posterior acoustic shadowing. **b** The bladder wall appears smooth and regular with respect to the urethra

The complete blood count (Bc5000vet, Shenzhen Mindray Bio-Medical Electronics Co.,Ltd., Shenzhen, China) results revealed an inflammatory leukogram with mild neutrophilia and monocytosis. Considering the history of disease and laboratory examination results, gastrointestinal or urinary system inflammation was preliminarily suspected. Then, cystography and abdominal ultrasound (a5vet, Dongguan Autrou Ultrasonic Technology Co.,Ltd., Dongguan, China) were performed to make a definitive diagnosis. The cystography was then performed as follows: the penis and prepuce were disinfected with a 0.02% chlorhexidine solution. A 1.3 French gauge, needle-free catheter was inserted into the urethral opening, carefully advancing it to reach the bladder. A total of 38 mL (8 ml/kg) of 20% iohexol (Iohexol injection, 15 g/50 ml, Zhejiang Tianrui Pharmaceutical Co.,Ltd., Zhejiang, China) was infused into the bladder and an abdominal radiography (e7240, Canon Inc, Tokyo, Japan) was taken immediately after withdrawing the urinary catheter. The CBC results are shown in Table 1. Cystography and abdominal ultrasound images are shown in Figs. 2 and 3, respectively.

**Table 1:** White blood cell counts of the cat from day 1 to day 16

Items	Day 1*	Day 3	Day 5	Day 10	Day 16	Reference
WBC	11.34	41.62	25.82	20.25	11.49	5.50 × 10^9^~19.50 × 10^9^/L
Neu#	9.97	40.25	24.79	19.14	7.53	3.12 × 10^9^~12.58 × 10^9^/L
Lym#	1.08	0.71	0.68	0.69	2.99	0.73 × 10^9^~7.86 × 10^9^/L
Eos#	00.6	0.00	0.07	0.16	0.31	00.6 × 10^9^~1.93 × 10^9^/L
Neu%	87.9	96.7	96.0	94.5	65.5	38.0 ~ 80.0%
Lym%	9.5	1.7	2.6	3.4	26.0	12.0 ~ 45.0%
Eos%	0.6	0.0	0.3	0.8	2.7	1.0 ~ 11.0%

*Note* * represents the day of surgery. WBC is the number of white blood cells; Neu# is the number of neutrophils; Lym# is the number of lymphocytes; EOS# is the eosinophil count; Neu% is the percentage of neutrophils; Lym% is the percentage of lymphocytes; and EOS% is the percentage of eosinophils. Other elements of the complete blood test results are not listed in the reference range

**Fig. 4:**
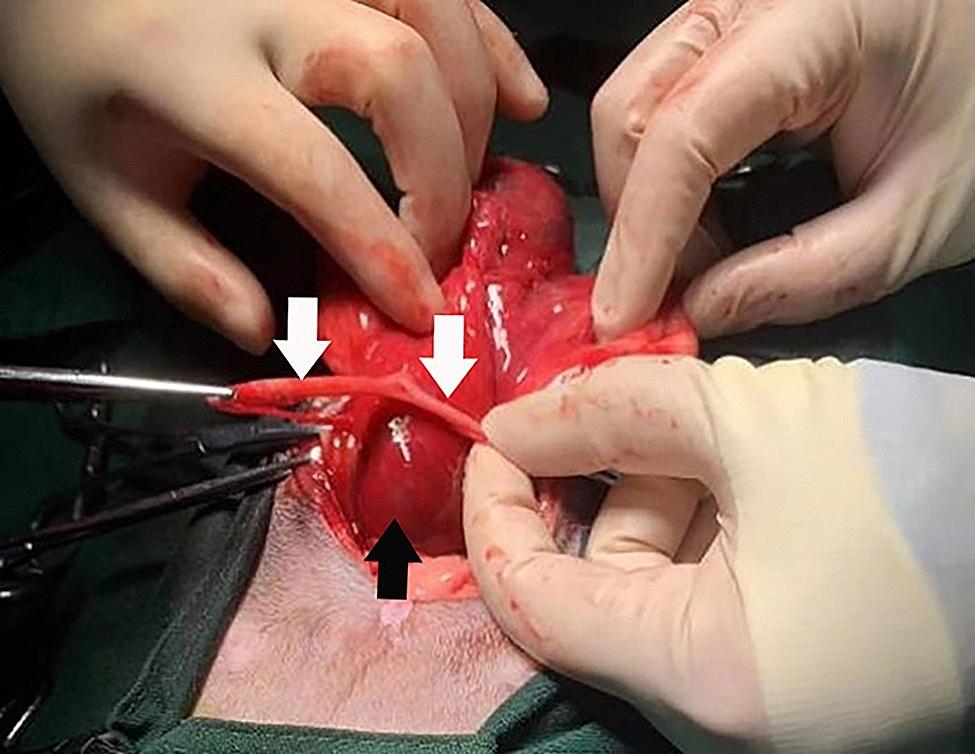
Image of the isolated cavitary structure of the cat. The two tubular tissues (white arrows) were pulled by the fingers and the hemostat, and the base of the tubular tissues was the cavitary structure (black arrows)

Guided by ultrasound, the cavitary structure was aspirated through the abdominal wall. Cytology of the aspirated fluid was examined with standardized slides preparation and then stained with Diff-Quik. Upon subsequent microscopic examination (CX23, Olympus Corporation, Tokyo, Japan), degenerative neutrophils were found on the slides. So purulent fluid was confirmed with cytological examination. Abdominal exploration was then performed. Preoperative anaesthesia was administered using 0.01 mg/kg medetomidine for sedation and analgesia. The procedure began with endotracheal intubation, Isoflurane (R510-22-10, RWD Life Science, Shenzhen, China) was used to maintain inhalation anesthesia (R650 Veterinary Anesthesia Machine, RWD Life Science, Shenzhen, China), with an oxygen flow rate of 1.5 L/min. The surgical site was shaved and disinfected. The surgical approach involved a midline abdominal incision extending from the umbilicus to the pubic bone that was approximately 5 cm in length. The bladder was not found in the proper anatomical location in the ventral abdomen. The surgeon extended the incision cranially, and a bladder-like cystic structure was found in the middle of the abdomen. The cystic structure was confirmed to be the bladder with a fine needle aspiration of urine. Another cavitary structure was then found on the dorsal distal side of the bladder that was soft in texture, bright red, and adhered to the surrounding hypertrophic tissues close to the subaortic artery. Two tubular tissues protruded from the top of the cavitary structure connected to the sides of the bladder. The purulent fluid was aspirated from the cavitary structure, which correlated with the anechoic structure found on ultrasound. There were no discernible large vascular structures with direct connections to the cavitary structure. The blood supply of cavitary structure is predominantly provided by the surrounding capillaries. The surgeon gently separated the cavitary structure from the surrounding tissues and resected it successfully. The dimensions of the cavitary tissue were 4.8 cm × 4.7 cm × 2.7 cm, and the two tubular structures measured 11.5 cm each. The cavitary structure with the accessory tubules can be seen in Fig. 4.

Postoperatively, the cat received 30 mg/kg ceftriaxone (1 g, Shenzhen Lijian Pharmaceutical Co., Ltd., Shenzhen, China) intravenously for three consecutive days for antimicrobial purposes. When the cat was rechecked on day 16, no inflammatory response was found and the urinalysis had returned to normal. The cat had a normal appetite and normal activity level with no clinical signs in the lower urinary tract.

**Fig. 5:**
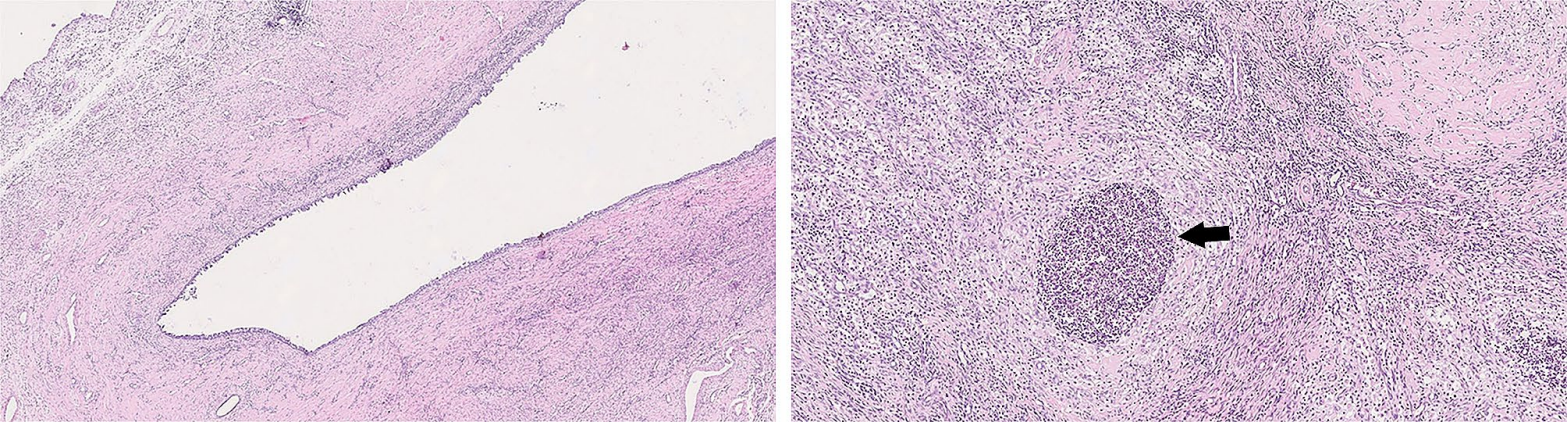
Histopathological images. On the left is a cavitary lesion containing neutrophils lined by transitional epithelium devoid of cellular atypia (urothelial epithelium); on the right is a detailed image of the inflammatory process involving neutrophils surrounded by macrophages. Within the lesion (black arrow), inflammatory infiltrates composed of aggregates of degenerated neutrophilic granulocytes surrounded by macrophages were abundant

Histopathological diagnosis was performed on the excised cavitary tissue. Histopathological slides were prepared and stained using hematoxylin and eosin (H&E). Microscopic examination of the tissues revealed severe pyogranulomatous cystitis and reactive fibroplasia. Histopathological results confirmed that the resected tissue was highly suspected to be a urinary bladder duplication. Histopathological images are shown in Fig. 5.

Based on the comprehensive evaluation of the disease history, physical examinations, laboratory examinations, imaging examinations, cytological examinations, and histopathological examination results, the disease was confirmed as feline bladder duplication.

## Discussion

**Fig. 6:**
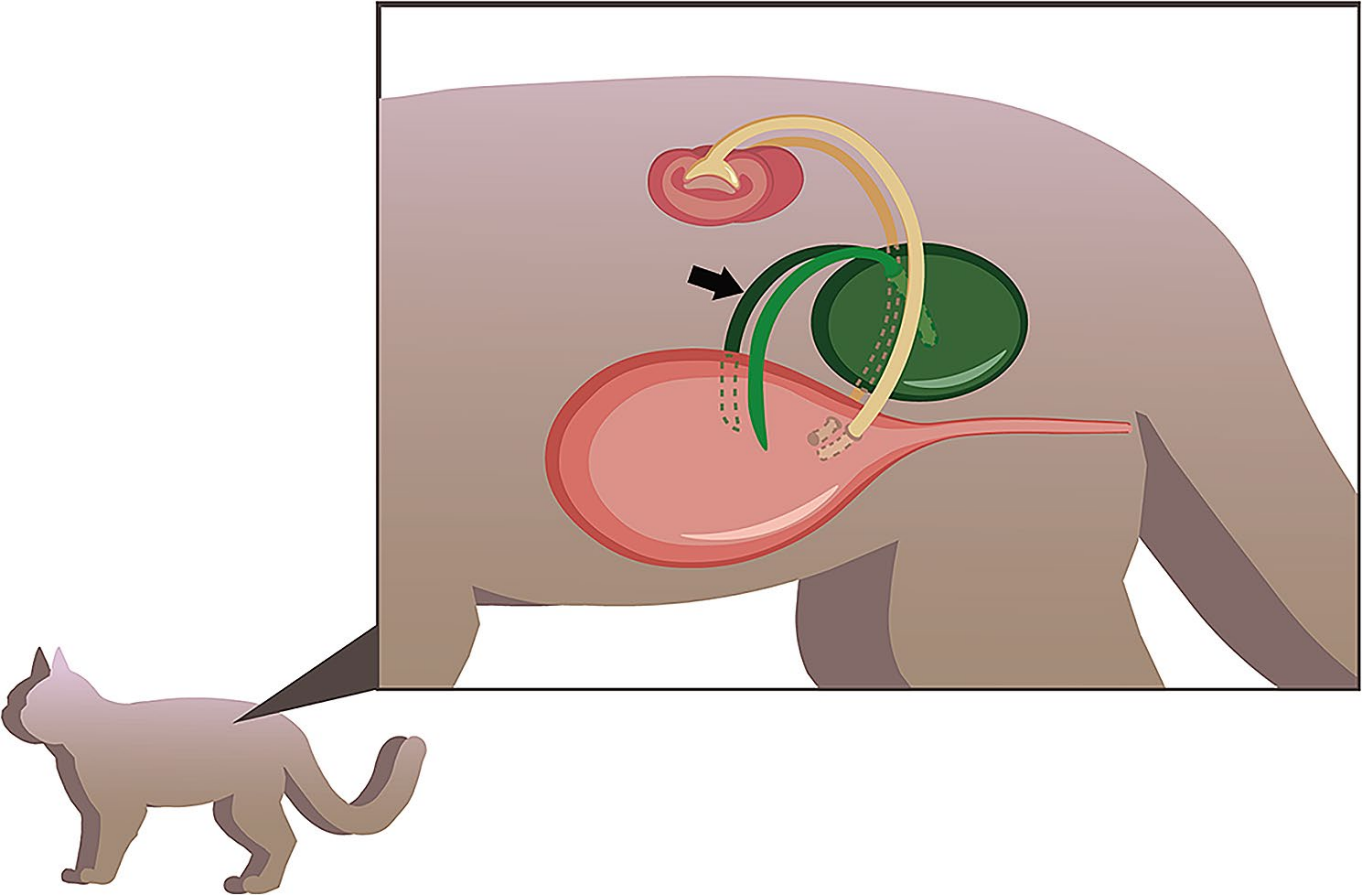
Diagram of bladder duplication in a cat. The pink region of the Hermosa ovary is the primary bladder, and the green region is the accessory bladder and the attached tubular tissue (arrow)

Bladder duplication (BD) is a rare congenital anomaly that occurs mostly in infancy and childhood in both humans and companion animals [4, 9]. Currently, the literature on BD in humans is limited to case reports and case series with no more than 90 cases since 2015 [4, 10]. For companion animals, cases reported in cats and dogs are extremely rare, with only one case in cats and 12 cases in dogs illustrating BDs and other urogenital duplications. In this case, the cat was diagnosed with complete bladder duplication with two accessory tubular tissues according to Abrahamson [11]. Complete duplication has been reported in dogs and one cat [5, 12–14]. In our patient, the morphology of the accessory bladder was an obstructed chamber, which was joined to the primary bladder by tubular tissue. The tubular tissue with one side tightly connected to the accessory bladder and the other side of the tube branched off and attached to the dorsal neck of the primary bladder (Fig. 6).

The pathogenesis of BD is still unclear. Researchers have investigated this kind of urogenital anomaly in humans since abnormal primordia develop during the foetal period. The theory is that ‘twinning of the embryo tail’ conceivably refers to the concurrence of duplicated bladders and other duplications, such as colons and genitalia, in the presence of a normal vertebral column [6]. Another theory given by Satter and Mossman, named the “double allantoic duct” [7], explains that the anomaly is initiated by the urogenital sagittal septum, doubling the original endodermal allantois. However, neither of the two classical theories implies that skeletal duplication relatively rarely occurs in BD patients [6, 7, 15, 16]. Only the embryogenesis of other duplicated organs is illustrated. The development of uterine didelphys is due to the failure of the fusion of paramesonephric structures, the Mullerian and Wolffian tubes [8]. It is known that ureteral sprouts also originate from Wolffian tubes [17], which suggests a possible association between the development of the urinary system and the ‘Mullerian ducts and Wolffian tubes system’. The generation of the urinary system is highly associated with the reproductive system, and therefore a high incidence of reproductive anomalies associated with BDs (85%) has been reported in humans [6, 18, 19]. Statistically, 40–56% of BD patients have colon duplication and other anorectal anomalies. Nearly 90% of female BD patients have duplicated vaginas. Approximately 10–15% of BD patients tend to have spinal malformations [15]. A dog was diagnosed with bladder duplication along with duplication of the urethra, cervix, and vaginal canal [9]. It was also observed in a cat with posterior duplication of the alimentary and urogenital system as well as the vertebral column and spinal cord [16]. In this case, the tubular tissues were associated with anomalies, which simultaneously occurred in BD patients. Moreover, the history of right undescended testis in our patient was similar to that of cryptorchidism in BD patients, both humans and dogs [6, 13, 14].

In the case of our patient, vomiting and constipation were the only clinical symptoms. Without further imaging diagnostic tools, we were unable to diagnose BD and other urogenital anomalies. As in human BD cases, the clinical signs of BD are usually nonspecific if no underlying infection occurs. In many cases, it is only recognized and diagnosed when an external anomaly such as duplicated genitalia or anus is present. An 18-month-old girl who presented with external anomalies of a cloacal exstrophy variant with an imperforate anus was subsequently diagnosed with BD by computer tomography (CT) [20]. In another case, the patient was a 4-year-old boy with a chronic history of intermittent abdominal pain and stunted growth for 2 years. He was not initially diagnosed with any specific disease until an advanced imaging diagnostic tool was used [21]. Generally, BD has no pathognomonic or specific signs, and for a majority of the time, it can be a disease of incidental diagnosis both in humans and companion animals.

Congenital urogenital malformations can be hard to diagnose, and special investigations are commonly performed to establish anomalies. In fact, various diagnostic methods have been used in human medicine for further study of congenital urinary anomalies [4, 20, 22]. Some of these materials have been used in veterinary medicine. Many radiographic techniques for diagnosing BD patients in humans and companion animals have been reported. Cystography is a contrast radiographic technique that uses X-rays or CT to assist in the detection of abnormal bladder morphology in cats [23]. A female cat with complete BD underwent negative contrast cystography to determine the location of the bladder and its integrity [5]. It further identifies whether the bladder is duplicated or the ureter is dilated [5]. Cystoscopy is applied to effectively assess the vagina, urethral opening, urethra, bladder, and ureteral openings in most cats [24]. In humans, cystoscopy was performed on urethras to determine the BD [7, 15, 22]. However, it has not been utilized to diagnose BD in animals before this case. Moreover, vaginocystourethrograms are helpful for diagnosing BD caused by duplication of the urethra, cervix, and vaginal canal in both humans and companion animals [5, 9]. Voiding cystourethrography (VCUG) is most often used to diagnose BD and several other urogenital anomalies in human infants and children [4, 20]. Due to the high morbidity of duplicated ureters in BD patients, subsequent intravenous urography (IVU) of BD patients is recommended for further investigation [9, 11, 16]. IVU has been applied in cats with urinary incontinence to reveal the renal function and morphology of the ureter [5, 11, 25–27]. However, this technique has been largely replaced by CT urography in human medicine [28]. In our patient, we performed cystography to locate the urinary bladder, which was confirmed to be the primary bladder after exploratory laparotomy and histopathological diagnosis. We considered CT a preferable exam for the diagnosis of accessory bladder and blind ending tubular tissues. Because there is no way for contrast agent to enter the duplicated bladder, the duplication is not visualized. We suggest using more advanced diagnostic tools to provide veterinarians with a more comprehensive surgical plan.

The principle of BD treatment needs to be individualized to each patient, depending on the patient’s symptoms and associated conditions. In human patients, surgical treatment for those with complete bladder duplication should be postponed until the patients reach the age at which their bladders become more identifiable [4]. In general, the goal of surgical intervention is to optimize bladder function and drainage as well as minimize incontinence and the risk of infection. To date, the most common surgical treatment for complete BD is to excise the duplicated bladder. The open approach for excision has been the most common choice both for humans and for companion animals [22]. Laparoscopic excision of complete bladder duplication was applied on a 1-year-old male in 2018 [22], suggesting that this technique is a desirable minimally invasive approach utilized for complex urinary anomalies in children. For the affected cat, excision of the accessory bladder was properly implemented due to inflammation and compression of other organs. In this case, Ceftriaxone, a third-generation cephalosporin exhibiting broad-spectrum antimicrobial properties, was utilized for the prevention of postoperative infections. The CBC results demonstrated that white blood cells and neutrophils counts return to within a normal range at day 16 post surgery, which means even with the systemic antibiotics, the inflammation response took two weeks to totally recover.

The prognosis depends on several factors, including the degree of obstruction, where the anomaly has been impaired, comorbidities, and secondary infections [11]. Canine patients have a good prognosis if they do not have any other skeletal or urogenital anomalies identified and if surgical corrections are to be performed [9].

## Conclusion

The number of similar cases in companion animals is extremely inadequate for reviewing the frequent occurrence of different duplicated urogenital organs and their pathogenesis. Logically, it is likely a heritable and genetic disease resulting from bladder duplication in companion animals. Thus, the author recommended surgical sterilization in dogs and cats with BD and accessory anomalies for disease control. Further genetic studies are warranted to understand the pathogenesis of this disease. It is essential that we record a case of bladder duplication with tubular tissues in a male cat; to the best of our knowledge, this is the first case of this type of malformation. As more BD cases are collected, more situations will be taken into consideration, and better advanced diagnosis and treatments can be assessed for patient benefit.

## Data Availability

Data is provided within the manuscriopt.

## Abbreviations

BD: Bladder duplication
BCS: Body condition score
CBC: Complete blood count
H&E: Haematoxylin and eosin
WBC: White blood cell
Neu: Neutrophils
Lym: Lymphocytes
EOS: Eosinophil
CT: Computer tomography
VCUG: Voiding cystourethrography
IVU: Intravenous urography
